# Illuminating stomatal responses to red light: establishing the role of *C*_i_-dependent versus -independent mechanisms in control of stomatal behaviour

**DOI:** 10.1093/jxb/erae093

**Published:** 2024-03-05

**Authors:** Georgia Taylor, Julia Walter, Johannes Kromdijk

**Affiliations:** Department of Plant Sciences, University of Cambridge, Cambridge CB2 3EA, UK; Department of Plant Sciences, University of Cambridge, Cambridge CB2 3EA, UK; Department of Plant Sciences, University of Cambridge, Cambridge CB2 3EA, UK; Carl R Woese Institute for Genomic Biology, University of Illinois at Urbana-Champaign, IL 61801, USA; University of Essex, UK

**Keywords:** Conductance, guard cells, intercellular CO_2_ concentration (*C*_i_), photosynthesis, red light, signalling, stomata

## Abstract

The stomatal response to red light appears to link stomatal conductance (*g*_s_) with photosynthetic rates. Initially, it was suggested that changes in intercellular CO_2_ concentration (*C*_i_) provide the main cue via a *C*_i_*-*dependent response. However, evidence for *C*_i_-independent mechanisms suggests an additional, more direct relationship with photosynthesis. While both *C*_i_-dependent and -independent mechanisms clearly function in stomatal red light responses, little is known regarding their relative contribution. The present study aimed to quantify the relative magnitude of *C*_i_-dependent and -independent mechanisms on the stomatal red light response, to characterize their interplay and to assess the putative link between plastoquinone redox state and *C*_i_-independent stomatal responses. Red light response curves measured at a range of *C*_i_ values for wild-type Arabidopsis (Col-0) and the CO_2_ hyposensitive mutant *ca1ca4* allowed deconvolution of *C*_i_-dependent and -independent pathways. Surprisingly, we observed that both mechanisms contribute equally to stomatal red light responses, but *C*_i_-independent stomatal opening is suppressed at high *C*_i_. The present data are also consistent with the involvement of the plastoquinone redox state in coordinating the *C*_i_*-*independent component. Overall, it seems that while *C*_i_-independent mechanisms are distinct from responses to *C*_i_, interplay between these two pathways is important to facilitate effective coordination between *g*_s_ and photosynthesis.

## Teaser

*C*_i_ -dependent and -independent pathways contribute equally to the stomatal red light response. Interaction between the pathways, which coordinate stomatal opening and closing, causes *C*_i_ -independent opening to be suppressed at high *C*_i_ .

## Introduction

Stomata are microscopic pores in the leaf epidermis that govern the pathway of carbon dioxide and water vapour exchange between plants and the surrounding atmosphere. Highly specialized guard cells (GCs) surround each pore and respond to various environmental and endogenous cues, such as light, CO_2_, humidity, and abscisic acid, to regulate stomatal conductance (*g*_s_) via changes in GC turgor pressure (Roelfsema *et al*., 2002; Inoue and Kinoshita, 2017; Jezek and Blatt, 2017; Lawson and Matthews, 2020). As such, GC responses are crucial for balancing photosynthetic demand for carbon with the need to conserve water (Wong *et al.*, 1979; Buckley, 2017). However, some of the exact mechanisms by which *g*_s_ and net CO_2_ assimilation (*A*_net_) are coordinated remain unclear.

Since light intensity and spectral quality are important determinants of *A*_net_, effective coordination between *g*_s_ and *A*_net_ requires *g*_s_ to respond to the prevailing light conditions. Stomatal responses to light can be divided into two distinct wavelength-dependent pathways. Blue light is perceived directly at the guard cells via two blue light photoreceptor proteins called phototropins (PHOT1 and PHOT2), inducing a well-characterized signal transduction cascade that leads to rapid membrane hyperpolarization and stomatal opening even at low fluences (Shimazaki et al., 1986, 2007; Kinoshita *et al.*, 2001; Takemiya *et al.*, 2013; Inoue and Kinoshita, 2017). By contrast, much less is known about the mechanisms that underpin stomatal responses to red light. Also known as the ‘quantitative’ light response, the red light response is slower than the response to blue light and occurs at higher irradiances, saturating at similar light intensities to photosynthesis (Sharkey and Raschke, 1981). The abolishment of red-light-induced stomatal opening by photosynthetic inhibitors, such as 3-(3,4-dichlorophenyl)-1,1-dimethylurea (DCMU) (Sharkey and Raschke, 1981; Olsen *et al.*, 2002; Messinger *et al.*, 2006; Wang *et al.*, 2011; Ando and Kinoshita, 2018), suggests stomatal red light responses are directly impacted by signals derived from photosynthesis (Lawson *et al.*, 2008; Matthews *et al.*, 2020). However, the exact location and mechanisms by which these putative photosynthesis-related signals arise and are communicated from to inflict changes in GC turgor remain elusive.

Early work suggested that stomatal responses to intercellular CO_2_ concentration (*C*_i_) provide the main cue for red light-induced stomatal opening, indirectly coupling *g*_s_ with the mesophyll’s demand for CO_2_ (Roelfsema *et al.*, 2002, 2006). Recent genetic advances have provided insight into the molecular mechanisms underlying this so-called ‘*C*_i_*-*dependent response’. The *C*_i_-dependent response can be divided into two CO_2_-concentration-dependent pathways, in which stomatal closure is promoted by elevated *C*_i_, whereas reductions in *C*_i_ provide a potent cue for stomatal opening (Mott, 1988; Fujita *et al.*, 2013; Engineer *et al.*, 2016; Dubeaux *et al.*, 2021). Within these opposing *C*_i_-dependent responses, multiple pathways exist and converge to modulate GC behaviour. Low *C*_i_ promotes stomatal opening via the RAF-like MAP kinase kinase kinase HIGH LEAF TEMPERATURE1 (HT1), which negatively regulates stomatal closure (Hashimoto *et al.*, 2006; Matrosova *et al.*, 2015; Hashimoto-Sugimoto *et al.*, 2016). Although the exact signalling cascade downstream of HT1 is not fully elucidated, two RAF-like kinases, CONVERGENCE OF BLUE LIGHT (CBC) 1 and 2, appear to be involved (Matrosova *et al.*, 2015; Hiyama *et al.*, 2017). Active HT1 has been shown to interact with and phosphorylate CBC1/2 *in vitro* (Hiyama *et al.*, 2017), and it is proposed that following phosphorylation, active CBC1/2 inhibit guard cell S-type anion channels, such as SLAC1, to suppress stomatal closure (Matrosova *et al.*, 2015; Hiyama *et al.*, 2017). Low CO_2_ also induces the phosphorylation and activation of plasma membrane H^+^-ATPases (Kinoshita and Shimazaki, 1999, 2002; Kinoshita *et al*., 2001), which in turn promote stomatal opening via activation of voltage-gated inward-rectifying K^+^ channels (Schroeder *et al.*, 1987).

By contrast, high *C*_i_ is perceived within the guard cells via two β-carbonic anhydrases, CARBONIC ANHYDRASE (CA) 1 and CA4, which strongly accelerate the equilibration between CO_2_ and bicarbonate (HCO_3_^−^) (Hu *et al.*, 2010; Xue *et al.*, 2011). In Arabidopsis, high CO_2_-induced stomatal closure is suppressed in plants lacking both enzymes and could be restored by complementation with CA1, CA4, or an unrelated mammalian αCAII (Hu *et al.*, 2010), suggesting that the catalytic activity of these CAs is required for high-CO_2_-induced stomatal closure. In addition, the MAP kinases MPK4 and MPK12 were recently identified as downstream CO_2_/HCO_3_^−^ sensors (Tõldsepp *et al.*, 2018; Takahashi *et al.*, 2022; Yeh *et al.*, 2023). In response to elevated HCO_3_^−^, MPK4/12 directly interact with and inhibit HT1, which in turn relieves HT1-mediated repression of GC S-type anion channels and promotes stomatal closure (Takahashi *et al.*, 2022; Yeh *et al.*, 2023). Concurrent with the HT1-mediated pathway of stomatal closure, high CO_2_ also promotes rapid dephosphorylation and deactivation of plasma membrane H^+^-ATPases, which suppresses stomatal opening (Edwards and Bowling, 1985; Ando *et al.*, 2022).

While changes in *C*_i_ provide a potent cue for stomatal opening and closing, an increasing body of evidence suggests that the stomatal red light response may also be controlled by *C*_i_-independent mechanisms. In particular, stomata continue to respond to changes in red light intensity when *C*_i_ is kept constant, suggesting alternative red light sensing and signalling pathways must also be involved (Messinger *et al.*, 2006; Lawson *et al.*, 2008; Wang and Song, 2008; Bernardo *et al.*, 2023). The maintenance of red light-induced stomatal opening in the CO_2_ hyposensitive mutant *ca1ca4* provides further evidence for a distinct *C*_i_-independent mechanism (Matrosova *et al.*, 2015; Ando *et al.*, 2022). Based on a synthesis of experimental work, Busch (2014) proposed that the redox state of the chloroplast plastoquinone (PQ) pool could provide an early photosynthesis-derived signal to coordinate stomatal responses with red light intensity. This putative relationship between PQ redox and *g*_s_ appears consistent with the altered stomatal responses observed in *Nicotiana tabacum* plants with modified levels of Photosystem II Subunit S (PsbS; Głowacka *et al*., 2018). Additionally, incorporation of the hypothesized PQ redox signal improved performance of models for stomatal conductance (Kromdijk *et al*., 2019). However, the mechanisms by which these redox signals could be communicated away from the chloroplast and ultimately coordinate stomatal behaviour are not yet understood.

While there is evidence for the involvement of both *C*_i_*-*dependent and -independent mechanisms in coordinating the stomatal response to red light, several open questions remain. For example, how much does each mechanism (i.e. *C*_i_*-*dependent and *C*_i_-independent) contribute to red light-induced stomatal opening? And are these pathways distinct or do they show interaction with one another?

In the present work, the objectives were to quantify the relative magnitude of *C*_i_-dependent and -independent mechanisms in coordinating the stomatal red light response, to characterize their interplay across a wide range of light intensities and *C*_i_, and to interrogate the relationship between PQ redox state and *C*_i_-independent stomatal movements across an extensive range of CO_2_ concentrations and light intensities. To decouple stomatal control via *C*_i_-dependent and -independent pathways, red light response curves were measured at a range of *C*_i_ values on wild-type Arabidopsis (Col-0) and on the CO_2_ hyposensitive mutant *ca1ca4*, which allowed further deconvolution of these pathways. Somewhat surprisingly, the results indicate that the contributions of *C*_i_-dependent and -independent mechanisms are similar in magnitude. Additionally, while the *C*_i_-independent response was found to be distinct from and additive to *C*_i_-dependent mechanisms of stomatal control, at high *C*_i_, *C*_i_-dependent closure is dominant and strongly suppresses *C*_i_-independent opening. The present data also support the hypothesized relationship between PQ redox state and *g*_s_. Overall, these results provide further insight into the mechanisms that control stomatal responses to red light and provide new avenues for future work to fully elucidate the molecular regulation involved.

## Materials and methods

### Plant materials and propagation

Seeds of Arabidopsis carbonic anhydrase knockout mutant *ca1ca4* (Hu *et al.*, 2010) were obtained from the Nottingham Arabidopsis Stock Centre (NASC) and genotyped via PCR (for primers see Supplementary Table S1). Col-0 and *ca1ca4* seeds were sown on a 4:1 mix of Levington® Advance F2 compost and sand and were stratified at 4 °C for 4 d. Seedlings were then transferred into individual 7×7 cm pots and positioned into a controlled growth chamber with a short-day photoperiod (8 h light–16 h dark). Light intensity was controlled at ~200 µmol m^−2^ s^−1^, relative humidity at 60%, and air temperature at 20 °C. Plants were hand-watered and randomly repositioned on the growth shelf every 3–4 d. All gas exchange measurements were performed on young fully expanded leaves of 7- to 9-week-old plants.

### Gas exchange measurements

#### Determining C_i_-dependent versus -independent responses

Col-0 or *ca1ca4* plants were first dark adapted for 2 h at ambient CO_2_, after which a fully expanded leaf (rosette leaf number 7–9) was clamped into the cuvette of an open gas exchange system (LI6400XT, LI-COR) with a 2 cm^2^ integrated fluorometer head (Leaf Chamber Fluorometer, LI6400-40, LI-COR). Block temperature was controlled at 25 °C, reference CO_2_ at 410 ppm, flow rate at 200 µmol s^−1^, and vapour pressure deficit (VPD) was maintained at ca. 1.1 kPa (±0.01 SE). The reference CO_2_ was then adjusted to obtain a defined *C*_i_ (75, 150, 300, 375, 450, 600, 750 µmol mol^−1^). Inside the cuvette, leaves were allowed to acclimate in the dark to the specified *C*_i_ until *g*_s_ had stabilized (taking at least an additional 30 min). The measuring light was then switched on briefly and once *F’* stabilized, chlorophyll fluorescence and gas exchange parameters were recorded. All fluorescence measurements were obtained using the multiphase flash routine (Loriaux *et al*., 2013), with the saturating flash set to 4000 µmol m^−2^ s^−1^ and a ramp of 30%. The minimal (*F*_o_) and maximal (*F*_m_) fluorescence were used to determine the maximal efficiency of whole-chain electron transport (*F*_v_/*F*_m_). The actinic red light, with a centre wavelength of 630 nm (±10 nm full width at half-maximum), was then increased stepwise: 0, 50, 100, 200, 400, 600, 800 µmol m^−2^ s^−1^. For each increase in actinic red light, the CO_2_ concentration of the cuvette was altered via the millivolt signal to maintain a constant *C*_i_ across the red light response curve. Plants were acclimated for at least 30 min at each light intensity and *C*_i_ before recording gas exchange and chlorophyll fluorescence parameters (*Fʹ*, *F*_o_ʹ, and *F*_m_ʹ). Table 1 provides the definitions and equations for the fluorescence parameters used. *F*_o_ʹ was estimated via the application of a weak pulse of far-red light (735 nm, ~10 µmol m^−2^ s^−1^) combined with a brief dark pulse (6 s) to fully oxidize the PQ pool. For leaves that did not completely fill the cuvette, an image of the leaf inside the gasket was taken and leaf area was calculated using ImageJ (National Institutes of Health, MD, USA) and adjusted in the gas exchange calculations. To account for diffusional leakage of CO_2_ from the cuvette, all gas exchange data were leak-corrected as per McDermitt *et al.* (1989) with some modifications. Briefly, leakage coefficients were calculated for each genotype using the dark-measured *A*_net_ values recorded for each *C*_i_ value. Gas exchange data (*A*_net_ and *C*_i_) were then corrected using these coefficients (McDermitt *et al.*, 1989).

**Table 1.** Definitions and equations of measured and calculated fluorescence parameters (Maxwell and Johnson, 2000; Kramer *et al.*, 2004; Baker, 2008; Murchie and Lawson, 2013)

Parameter	Definition	Equation
*F*_o_	Minimal fluorescence (dark-adapted)	
*F*_m_	Maximum fluorescence (dark-adapted)	
*Fʹ*	Steady-state fluorescence	
*F*_o_ʹ	Minimal fluorescence (light-adapted)	
*F*_m_ʹ	Maximal fluorescence (light-adapted)	
*F*_v_/*F*_m_	Maximum quantum yield of PSII (dark-adapted)	(*F*_m_*−F*_o_) / *F*_o_
*F*_q_ʹ*/F*_m_ʹ (Φ_PSII_)	Operating efficiency of PSII	(*F*_m_ʹ*−Fʹ*) / *F*_m_ʹ
NPQ	Non-photochemical quenching	(*F*_m_*−F*_m_ʹ) / *F*_m_ʹ
1−*q*_L_	Q_A_ redox state	*q*_L_=(1/*Fʹ−*1/*F*_m_ʹ) / (1/*F*_o_ʹ*−*1/*F*_m_ʹ)

### Determining steady-state ‘physiologically relevant’ *C*_i_ values

Col-0 plants were dark adapted for 2 h, after which a fully expanded leaf (rosette leaf number 7–9) was clamped into the gas exchange cuvette. Block temperature was controlled at 25 °C, reference CO_2_ at 410 ppm, flow rate at 200 µmol s^−1^, and VPD was maintained at ca. 1.1 kPa. Actinic red light (100%) was increased in a stepwise manner across a physiologically relevant range of light intensities: 0 (dark; D), 50 (low-light; LL), 200 (growth-light; GL) and 800 (high-light; HL) µmol m^−2^ s^−1^. The *C*_i_ values obtained at these light intensities were used to determine a range of physiologically relevant *C*_i_ values.

### Statistical analysis

Two-way repeated measures ANOVA was used to assess the significance of effects of red light intensity, *C*_i_, and their interaction on stomatal conductance. Additionally, η^2^ was calculated to estimate the effect size associated with each dependent variable; in a repeated measures design η^2^ can be calculated using the following equation: η^2^=SS_effect_/(SS_effect_+SS_error_), where SS_effect_ is the sum of squares (SS) or variance associated with each effect, and SS_error_ is the variance that cannot be explained, i.e. the error variance. For each ANOVA, assumptions of normality, homogeneity of variance and sphericity were tested. For data that failed to meet these assumptions, a non-parametric Kruskal–Wallis rank sum was used, followed by Dunn’s multiple comparison test. The relationship between Q_A_ redox and stomatal conductance was analysed using multiple linear regression, and minimum models for Col-0 and *ca1ca4* were obtained via backwards stepwise elimination. All data analysis and plot generation were carried out using R 4.1.1 (R Core Team 2021) on RStudio (Posit Team, 2022).

## Results

### *C*_i_-dependent and -independent mechanisms contribute equally to the stomatal red light response

**Fig. 1.**
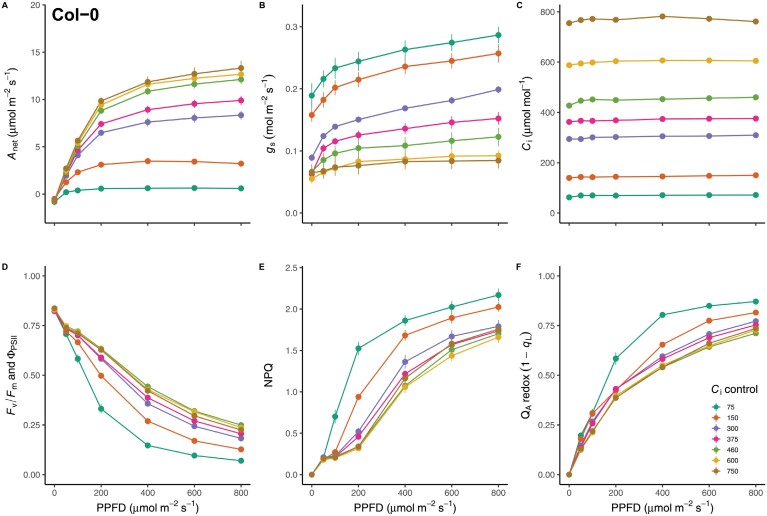
The photosynthetic response of wild-type (Col-0) Arabidopsis to increasing red light under a range of controlled intercellular CO_2_ (*C*_i_) values. (A–C) Leaf level gas exchange parameters CO_2_ assimilation (*A*_net_) (A) and stomatal conductance (*g*_s_) (B) were measured at a range of controlled *C*_i_ values (C). (D–F) Chlorophyll fluorescence was concomitantly measured after stabilization at each light level to probe the effect of *C*_i_ and light intensity on the response of the photosynthetic electron transport chain: quantum efficiency of PSII (Φ_PSII_) (D), non-photochemical quenching (NPQ) (E), and the redox state of quinone A (Q_A_) as estimated by the parameter 1−qL (F) (Kramer *et al.*, 2004). *n*=7. PPFD, photosynthetic photon flux density.

To empirically quantify the relative contributions of *C*_i_*-*dependent and *C*_i_*-*independent mechanisms to the stomatal red light response, gas exchange measurements were performed in Arabidopsis (Col-0) plants at a matrix of seven red light intensities and seven *C*_i_ values to obtain red light dose–response curves of *A*_net_ (Fig. 1A) and *g*_s_ (Fig. 1B) across *C*_i_ values spanning 75–750 µmol mol^−1^. To separate *C*_i_-dependent and *C*_i_-independent responses, *C*_i_ was kept constant within each red light response curve (Fig. 1C) by carefully adjusting the CO_2_ concentration of the cuvette after each change in light intensity, following the approach by Messinger *et al*. (2006). In this way, the response of *g*_s_ to red light intensity within each response curve can be taken as a direct measure of *C*_i_-independent mechanisms, while the separation between response curves at different *C*_i_ values provides an estimate of *C*_i_-dependent mechanisms.

As expected, *A*_net_ increased hyperbolically with increasing red light intensity. At *C*_i_ values of 75 and 150 µmol mol^−1^, *A*_net_ was strongly inhibited by limiting CO_2_ supply, whereas *A*_net_ reached saturation for *C*_i_ values above 460 µmol mol^−1^ (Fig. 1A). Interestingly, *g*_s_ values were strongly influenced by red light intensity changes at each constant *C*_i_ as well as by the different *C*_i_ levels between the response curves (Fig. 1B). Namely, while *C*_i_ was kept constant across each light response curve, *g*_s_ displayed a continued opening response to increasing red light intensity at each *C*_i_ level (Fig. 1B). Although the general shape of this opening response remained largely consistent between the different *C*_i_ values, the entire *g*_s_*–*light curves were transposed along the *g*_s_-axis, due to clear separation in initial (*g*_s,int_) and maximal (*g*_s,max_) *g*_s_ resulting from the contrasting *C*_i_ values. In line with these observations, two-way repeated measures ANOVA demonstrated highly significant effects of red light intensity at constant *C*_i_ (*F*(6,47)=321.93, *P<*0.001), *C*_i_ level (*F*(6,36)=39.67, *P*<0.001) and their interaction (*F*(36,282)=9.201, *P<*0.001) on the response of *g*_s_. Eta squared (η^2^) was calculated to estimate the effect size of each predictor variable, and their interaction. Remarkably, both light intensity (η^2^=0.87) and *C*_i_ (η^2^=0.84) had very similar effect size, with slightly smaller effect size of the interaction (η^2^*=*0.54). Interestingly, the similar η^2^ values obtained for light intensity and *C*_i_ suggest their influence on stomatal opening is similar in magnitude. The smaller, but still appreciable η^2^ value obtained for the interaction term can be explained by the suppression of light-induced stomatal opening at high *C*_i_, highlighting an interaction between *C*_i_-dependent and -independent mechanisms under specific conditions, such as at high *C*_i._

Changes in electron transport parameters were concomitantly estimated from fluorescence measurements taken alongside gas exchange after acclimation to each light level (Fig. 1D–F). The maximal efficiency of PSII after dark acclimation (*F*_v_/*F*_m_, Fig. 1D) was 0.83±0.001, similar across all measurements and was not significantly affected by *C*_i_ value (*P=*0.16). For *C*_i_ values above 150 µmol mol^−1^, the quantum efficiency of PSII (Φ_PSII_) as a function of red light intensity was similar, whereas low *C*_i_ values (<150 µmol mol^−1^) caused a reduction in Φ_PSII_ (Fig. 1D), consistent with carbon supply limiting CO_2_ assimilation (Fig. 1A) and consequently inhibiting linear electron flow. Likewise, carbon supply limitation also affected the NPQ response, where a greater induction of NPQ was observed at low *C*_i_ values (Fig. 1E). Plastoquinone redox state can be approximated by the fluorescence parameter 1−*q*_L_ (Kramer *et al*., 2004), which estimates the redox state of the first stable electron acceptor at PSII, the quinone bound to the Q_A_ site. In line with the observed patterns of Φ_PSII_, Q_A_ redox state as a function of red light intensity was similar for *C*_i_ values above 150 µmol mol^−1^, but lower *C*_i_ values caused the Q_A_ pool to become more reduced (i.e. higher values of 1−*q*_L_, Fig. 1F).

The wide range of controlled *C*_i_ values used to construct the red light response curves in Fig. 1C may exceed the *C*_i_ levels typically experienced by plants under steady-state non-stressed conditions. To assess the most relevant range of *C*_i_ values for red light stomatal responses in non-stressed Arabidopsis plants, a second set of light response measurements was performed. For these measurements, the CO_2_ concentration in the cuvette was kept at the approximate level during plant growth (410 µmol mol^−1^), allowing *C*_i_ values to respond to each change in light intensity (Supplementary Fig. S1C). Concomitant with increasing *A*_net_ and *g*_s_ (Supplementary Fig. S1A, B), *C*_i_ declined with increasing red light intensity (Supplementary Fig. S1C), ranging from 437±3 µmol mol^−1^ in darkness to 312±5 µmol mol^−1^ at a light intensity of 800 µmol m^−2^ s^−1^. We therefore estimate the physiologically relevant *C*_i_ range for stomatal control in these plants to be approximately between 300 and 450 µmol mol^−1^. As such, we reanalysed the relationship between stomatal conductance, *C*_i_, and light intensity using only curves at *C*_i_ values within this physiologically relevant range. A two-way ANOVA showed a significant effect of *C*_i_ (*P<*0.002), light intensity (*P*<0.001), and their interaction (*P<*0.001) on stomatal conductance. Interestingly, the effect size of these factors shifted, whereby the contribution of light intensity remained similar (η^2^=0.91), while the effect size associated with *C*_i_ (η^2^=0.43) and their interaction (η^2^=0.42) was reduced. This suggests that within a physiologically relevant *C*_i_ range, the quantitative actinic red light-specific opening response exceeds the response to *C*_i_.

### Stomatal conductance responds strongly to *C*_i_ in darkness

**Fig. 2.**
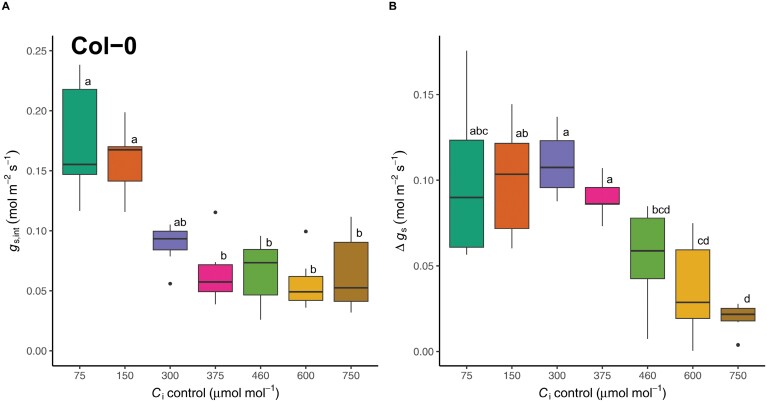
Quantifying the effect of intercellular CO_2_ concentration (*C*_i_) on the initial stomatal response in the dark (*g*_s,int_) (A), which was recorded after at least 30 min stabilization at each controlled *C*_i_, and the total stomatal opening response (Δ*g*_s_*=g*_s,max_–*g*_s,int_) (B). *n*=7.

Interestingly, different *C*_i_ values seemed to inflict substantial changes in *g*_s_ in the absence of light. Indeed, statistical analysis of *g*_s,int_, which was recorded after at least 30 min of acclimation to the controlled *C*_i_ in darkness (Fig. 2A), showed a significant effect of *C*_i_ value (Kruskal–Wallis *H*=36.129, df=6, *P<*0.001). In particular, sub-ambient *C*_i_ values induced a significant stomatal opening response, with approximately 3-fold higher *g*_s,int_ at *C*_i_ of 75 and 150 µmol mol^−1^, compared with any of the *C*_i_ values greater than 375 µmol mol^−1^ (*P*<0.05; for all Dunn’s comparisons, see Supplementary Table S2). These results clearly demonstrate that stomatal movements via *C*_i_-dependent mechanisms occur in darkness, and that low *C*_i_ values are sufficient to induce significant stomatal opening independently of light.

### The magnitude of *C*_i_-independent stomatal responses to red light is suppressed by high *C*_i_

To extract the response range of *C*_i_-independent responses to red light, Δ*g*_s_ (=*g*_s,max_–*g*_s,int_) was calculated for each *C*_i_ value (Fig. 2B). Δ*g*_s_ was significantly affected by *C*_i_ value (Kruskal–Wallis *H=*34.422, df=6, *P<*0.001). Δ*g*_s_ was approximately 0.1 mol m^−2^ s^−1^ and did not vary significantly for response curves measured at *C*_i_ values less than 460 µmol mol^−1^ (*P*<0.05; for all Dunn’s comparisons, see Supplementary Table S2), demonstrating that *C*_i_ values did not affect the magnitude of *C*_i_-independent mechanisms within this range. Interestingly, the most pronounced Δ*g*_s_ values were observed at *C*_i_ values within the physiologically active range, at 300 µmol mol^−1^. By contrast, a reduction in Δ*g*_s_ was observed in response to high *C*_i_ (>460 µmol mol^−1^), and the extent of Δ*g*_s_ inhibition increased with *C*_i_ values, such that the opening response associated with *C*_i_-independent mechanisms was almost completely abolished at a *C*_i_ value of 750 µmol mol^−1^.

### CO_2_ hyposensitivity maintains *C*_i_-independent stomatal opening across the full *C*_i_ range

**Fig. 3.**
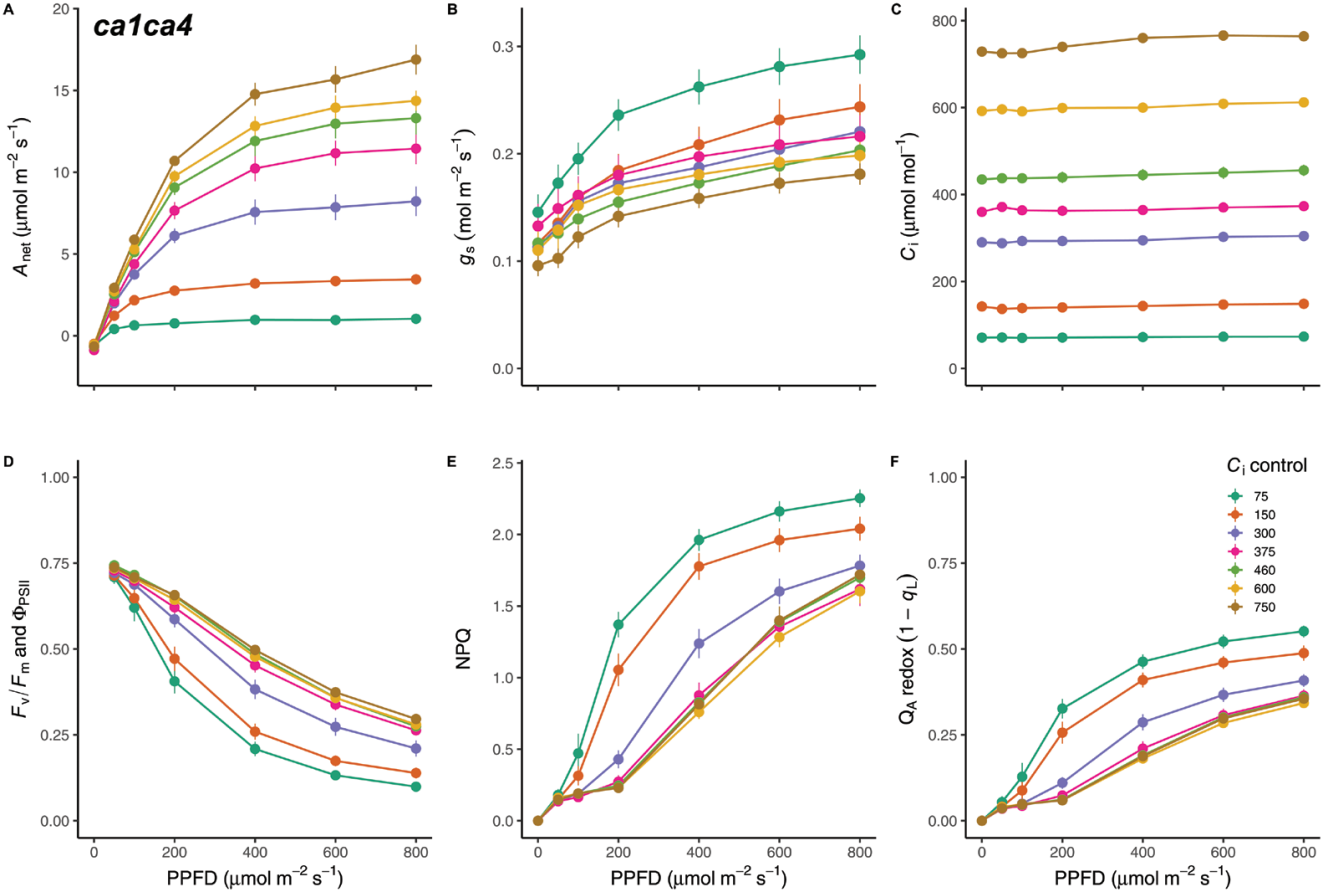
Intercellular CO_2_ concentration (*C*_i_)-dependent, but not *C*_i_-independent, stomatal responses are diminished in the CO_2_ hyposensitive mutant *ca1ca4.* (A–C) Leaf level gas exchange parameters CO_2_ assimilation (*A*_net_) (A) and stomatal conductance (*g*_s_) (B) recorded at a range of controlled *C*_i_ values (C). (D–F) The effect of light intensity and *C*_i_ value on the response of the photosynthetic electron transport chain was estimated using the chlorophyll fluorescence parameters quantum efficiency of PSII (Φ_PSII_) (D), non-photochemical quenching (NPQ) (E), and the redox state of quinone A (Q_A_) (F). *n*=7. PPFD, photosynthetic photon flux density.

To further investigate the observed suppression of *C*_i_-independent stomatal opening at high *C*_i_ in Col-0, responses of *A*_net_ (Fig. 3A) and *g*_s_ (Fig. 3B) to increasing red light under the same controlled *C*_i_ treatments (Fig. 3C) were measured on CO_2_ hyposensitive *ca1ca4* plants. As expected, *A*_net_ of *ca1ca4* increased with red light intensity and was strongly inhibited at low *C*_i_ and elevated above ambient at high *C*_i_ (Fig. 3A). Interestingly, while *ca1ca4* demonstrated a strong stomatal opening response to increasing red light (Fig. 3B), the *C*_i_-dependent separation of stomatal responses was not very pronounced. Instead, stomatal responses clustered together for all *C*_i_ values above 75 µmol mol^−1^ (Fig. 3B). Notably, a pronounced increase in *g*_s,max_ was observed at a *C*_i_ value of 75 µmol mol^−1^, indicating that the response to low *C*_i_ is at least partially maintained in this double mutant. Indeed, a two-way repeated measures ANOVA demonstrated a significant effect of *C*_i_ (*F*(5,45)=3.617, *P=*0.005) and a highly significant effect of red light intensity (*F*(6,36)=369.179, *P*<0.001), and their interaction (*F*(36,270)=3.558, *P*<0.001) on the stomatal response. Interestingly, when the proportion of variance associated with each variable in *ca1ca4* was calculated, the contribution of light intensity remained high (η^2^=0.89), while the effect sizes of *C*_i_ (η^2^=0.33) and the interaction (η^2^*=*0.16) decreased considerably due to the CO_2_ hyposensitivity in this genotype.

Carbon supply limitation influenced the responses of Φ_PSII_, NPQ, and Q_A_ redox state to light intensity (Fig. 3D–F). *F*_v_/*F*_m_ was not significantly affected by *C*_i_ value (*F*_v_/*F*_m_=0.831±0.002, *P=*0.292). However, for *C*_i_ of <300 µmol mol^−1^, a pronounced reduction of Φ_PSII_ was observed (Fig. 3D), with a concomitant increase in NPQ activation (Fig. 3E) and Q_A_ reduction (Fig. 3F).

### *C*_i_ *-*dependent responses are impaired in *ca1ca4*

**Fig. 4.**
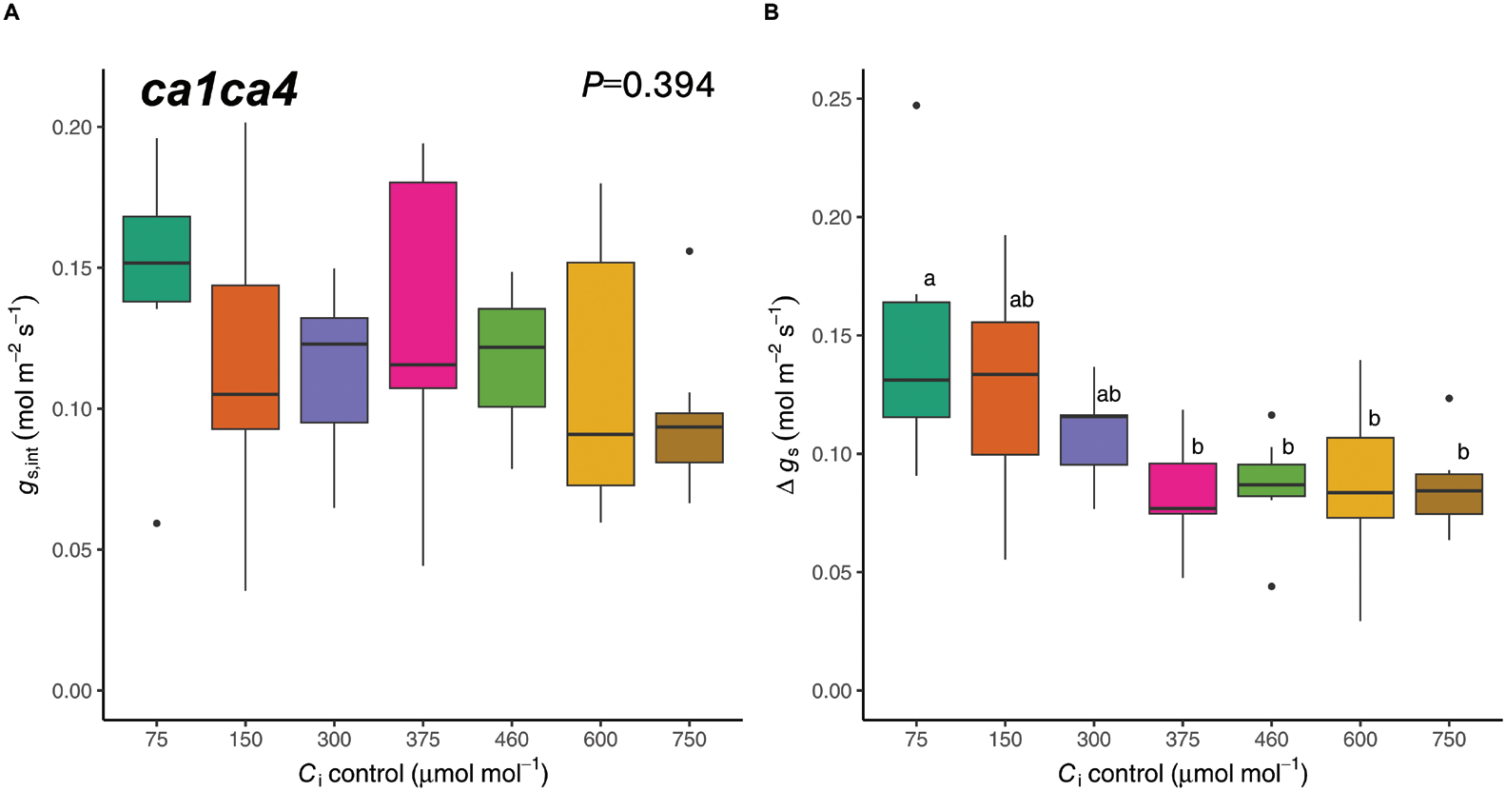
The effect of intercellular CO_2_ concentration (*C*_i_) on the initial stomatal conductance in the dark (*g*_s,int_) (A) and the magnitude of stomatal opening (Δ*g*_s_) (B) is abolished in the CO_2_ hyposensitive double mutant *ca1ca4. n*=7.

Interestingly, *C*_i_*-*induced stomatal movements under dark conditions (*g*_s,int_) were not significant in *ca1ca4* (Fig. 4A; *F*(6,45)=1.072, *P=*0.394), suggesting that the activity of CA1/CA4 is crucial for the sensing and coordinating of CO_2_-induced stomatal movements in the absence of light.

To investigate the effect of CO_2_ hyposensitivity on the magnitude of *C*_i_-independent stomatal opening, we also compared Δ*g*_s_ across all *C*_i_ values for *ca1ca4* (Fig. 4B). While Δ*g*_s_ was still significantly affected by *C*_i_ (*F*(6,45)=3.98, *P=*0.003), high *C*_i_-induced suppression of *C*_i_-independent stomatal opening was not observed for *ca1ca4* and Δ*g*_s_ was similar across all *C*_i_ values >75 µmol mol^−1^ (*P*<0.05; for all Tukey’s comparisons, see Supplementary Table S3).

### Testing the relationship between plastoquinone redox state and *C*_i_-independent red light responses

**Fig. 5.**
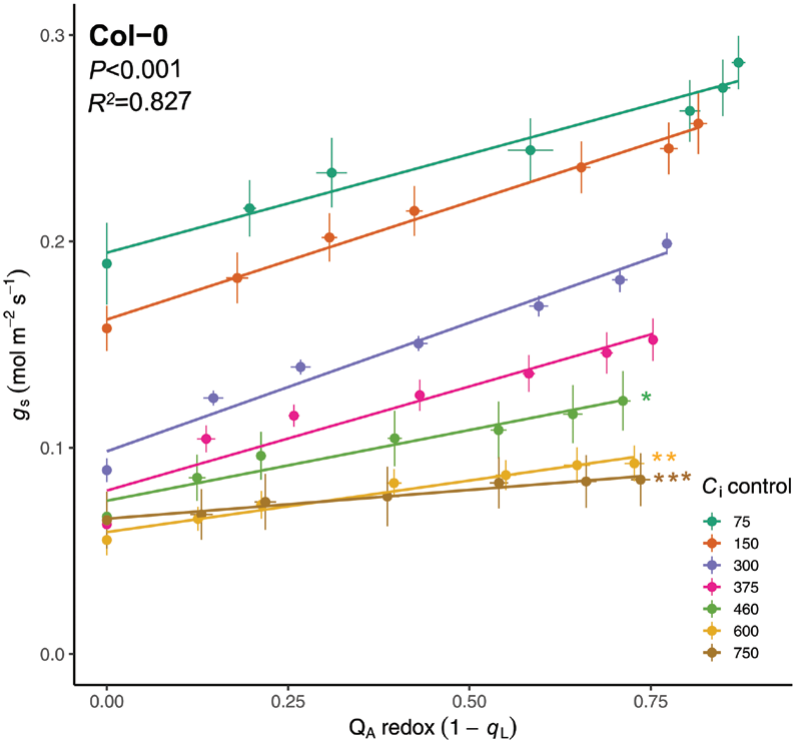
Multiple linear regression of the relationship between Q_A_ redox state and stomatal conductance (*g*_s_) under constant intercellular CO_2_ concentration (*C*_i_) shows suppression of *C*_i_-independent stomatal opening by high *C*_i_*-*induced stomatal closure. *n*=7.

To address the hypothesized relationship between PQ redox state and red light-induced stomatal movements, we analysed the relationship between 1−*q*_L_ (Q_A_ redox) and the *C*_i_-independent component of the red light response curves in Col-0 (Fig. 5). Interestingly, a more linear relationship was observed between Q_A_ redox state and *g*_s_ (Fig. 5) compared with the relationship between *g*_s_ and red light intensity (Fig. 1B), suggesting Q_A_ redox state could be a better predictor of *g*_s_. Multiple linear regression in combination with backwards stepwise elimination yielded a minimal model for prediction of *g*_s_ with significant main effects of Q_A_ redox state, *C*_i_, and their interaction, which was able to capture a significant proportion of the experimental variance (*R*^2^=0.827, *F*(13,364)=137.2, *P<*0.001; see Supplementary Table S3). The significant interaction between Q_A_ redox state and *C*_i_ value reflected the fact that at *C*_i_ values below 375 µmol mol^−1^ individual regression lines yielded similar slopes (Fig. 5A; Supplementary Table S3), while at *C*_i_ values above 460 µmol mol^−1^, slopes of the regression lines were significantly decreased (Fig. 5A; Supplementary Table S4), due to the observed stomatal closure response elicited by high *C*_i_, which counteracted the effect of *C*_i_-independent red light opening responses (Figs 1B, 2B). This suggests that at low *C*_i_ (<375 µmol mol^−1^), knowledge of Q_A_ redox state and *C*_i_ can be used to accurately predict *g*_s_, whereas at high *C*_i_ (>460 µmol mol^−1^), due to high *C*_i_-induced suppression of stomatal opening, knowledge of the interaction between Q_A_ redox state and *C*_i_ is also required.

**Fig. 6.**
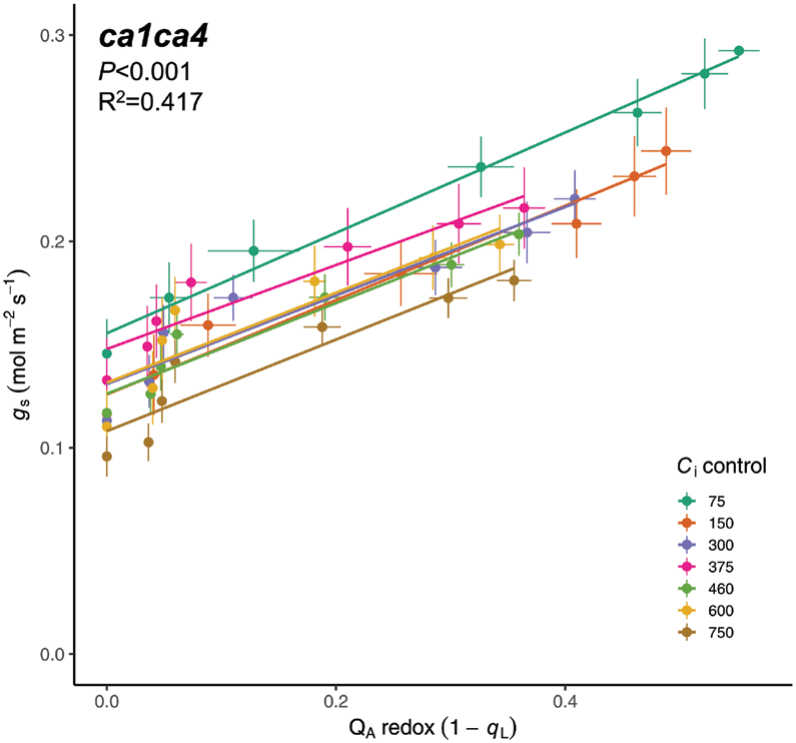
Multiple linear regression of the relationship between Q_A_ redox state and stomatal conductance (*g*_s_) in the CO_2_ hyposensitive mutant *ca1ca4. n*=7.

Intriguingly, the CO_2_ hyposensitivity of *ca1ca4* caused the positive relationship between Q_A_ redox state and *g*_s_ to be maintained across all measured *C*_i_ values (Fig. 6). As a result, multiple linear regression followed by backwards stepwise elimination provided a minimum model that did not include an interaction term, but only significant main effects of *C*_i_ and Q_A_ redox state on stomatal conductance (*R*^2^=0.417, *F*(7,356)=45.1, *P<*0.001; see Fig. 6; Supplementary Table S5). This indicates that in the absence of high *C*_i_-induced stomatal closure, the modelled effects of Q_A_ redox state and *C*_i_ on *g*_s_ become additive across the full range of *C*_i_ values, and as such, *g*_s_ can be predicted by a linear combination of Q_A_ redox state and *C*_i_.

## Discussion

In this work the contribution of *C*_i_-dependent and *C*_i_-independent mechanisms on the stomatal red light response was quantitatively assessed. By keeping *C*_i_ constant across red light dose–response curves at values spanning 75 to 750 µmol mol^−1^, including concentrations within a ‘physiologically active range’, we were able to decouple *C*_i_*-*dependent from *C*_i_-independent mechanisms of stomatal control. Subsequent analysis of these responses in the CO_2_ hyposensitive mutant *ca1ca4* allowed further deconvolution of the involvement of these two red light-associated mechanisms. For the first time, our results demonstrate that the influence of *C*_i_-dependent and *C*_i_-independent mechanisms are similar in magnitude. When the relationship between *g*_s_, *C*_i_, and red light intensity was focused to within a physiologically relevant *C*_i_ range (300–450 µmol mol^−1^; Supplementary Fig. S1), the contribution of the red light-specific *C*_i_-independent mechanism was considerably higher than the effect of *C*_i_. Additionally, the observed suppression of *C*_i_-independent stomatal opening at high *C*_i_ values provides evidence for crosstalk between these two pathways. These findings are discussed further in the following paragraphs.

### *C*_i_ *-*dependent and -independent mechanisms contribute equally to stomatal red light responses

The control of *C*_i_ over stomatal movements is well-known (Roelfsema *et al*., 2002, 2006) and increasing evidence suggests that both *C*_i_*-*dependent and -independent mechanisms coordinate the stomatal red light response (Messinger *et al.*, 2006; Lawson *et al.*, 2008; Wang and Song, 2008; Matrosova *et al.*, 2015; Bernardo *et al.*, 2023). However, quantitative knowledge regarding the proportional contribution and extent of crosstalk between *C*_i_-dependent and -independent pathways has so far remained limited. Somewhat surprisingly, our results demonstrate that *C*_i_*-*dependent and *C*_i_-independent mechanisms of stomatal control are approximately equal in magnitude (Fig. 2B). The experimental design employed here allowed easy separation of *C*_i_*-*dependent and -independent components. While the response of *g*_s_ to the controlled *C*_i_ value during each red light response curve confirmed the established role of *C*_i_-dependent stomatal movements (Roelfsema *et al*., 2002, 2006; Assmann and Jegla, 2016; Engineer *et al.*, 2016), stomata also continued to respond to increasing red light intensity when *C*_i_ was held constant across a range of values (Fig. 2B), clearly supporting a quantitative red light response that is independent of *C*_i_. Although evidence for *C*_i_-independent control of stomatal behaviour exists (Messinger *et al.*, 2006; Lawson *et al.*, 2008; Wang and Song, 2008; Bernardo *et al.*, 2023), such studies have controlled *C*_i_ at a single value, and as such, provide limited scope for interpreting the magnitude and coordination of *C*_i_-dependent and -independent mechanisms.

### Is there crosstalk between *C*_i_*-*dependent and -independent stomatal red light responses?

The present experimental design allowed the relationship between *C*_i_-dependent and -independent mechanisms to be interrogated across a wide range of *C*_i_ values. For the first time, we clearly show that for *C*_i_ <460 µmol mol^−1^, the effects of *C*_i_-dependent and -independent mechanisms on *g*_s_ are independent and additive (Figs 1B, 2, 5), whereas observations at higher *C*_i_ showed strong suppression of the *C*_i_-independent mechanism, suggesting that the molecular determinants of the closing response at high CO_2_ may intersect with those of the *C*_i_-independent opening response. The observed stomatal opening response to low *C*_i_ in darkness (Figs 1B, 2A), i.e. in the absence of a red light signal, provides further evidence that the *C*_i_*-*dependent mechanism is distinct from alternative red light-driven stomatal responses. While in previous work stomatal opening in response to CO_2_ in the dark was less pronounced and delayed (Sharkey and Raschke, 1981; Messinger *et al.*, 2006; Hiyama *et al.*, 2017; Hosotani *et al.*, 2021), here a substantial opening response was observed, such that at *C*_i_ values below 150 µmol mol^−1^, stomatal opening in the dark (*g*_s,int_) exceeded maximal stomatal conductance (*g*_s,max_) for measurements taken at *C*_i_ values above 300 µmol mol^−1^. This raises the question, which molecular mechanisms are required to drive the stomatal opening response to CO_2_ in darkness? Notably, low-CO_2_-induced stomatal opening in the absence of light was abolished in plants lacking HT1 and CBC1/CBC2 (Hiyama *et al.*, 2017), suggesting HT1–CBC-mediated suppression of S-type anion channels is required to facilitate stomatal opening in the dark. Interestingly, Hiyama *et al.* (2017) also showed that while stomata respond to low CO_2_ in the dark, no further stomatal closure was observed in response to high CO_2_ (800 µmol mol^−1^). This is consistent with the present study, as *g*_s,int_ was similar between all *C*_i_ >375 µmol mol^−1^, showing that stomata are already maximally closed under ambient CO_2_ in the dark. The threshold *C*_i_, below which the opening response to low *C*_i_ overrides competing pathways of dark-induced closure, was found to lie between 300 and 375 µmol mol^−1^, as evident from the intermediate *g*_s,int_ values observed at the former (Fig. 1A). Interestingly, the *C*_i_-independent response estimated by Δ*g*_s_ was unaffected by the initial dark conductance. For example, while the *g*_s,int_ measured at a *C*_i_ value of 75 µmol mol^−1^ was greater than the final *g*_s,max_ (at 800 µmol m^−2^ s^−1^) observed for *C*_i_ values above 300 µmol mol^−1^ (Fig. 1B), Δ*g*_s_ remained similar (Fig. 2A). We therefore postulate that the *C*_i_*-*dependent pathway functions as a ‘coarse’ baseline control of stomatal movements, whereas the *C*_i_-independent stomatal response to red light could be involved in further fine-tuning, to facilitate a more direct relationship with concurrent photosynthetic rates (Messinger *et al.*, 2006; Lawson *et al.*, 2008). In contrast to the independent operation of *C*_i_-dependent and -independent responses at low *C*_i_, the magnitude of Δ*g*_s_ was significantly suppressed under high *C*_i_ (Fig. 2B). Thus, high *C*_i_*-*induced stomatal closure is dominant and suppresses C_i_-independent stomatal opening. The observed competition between pathways is consistent with recent work where high CO_2_-mediated dephosphorylation of guard cell plasma membrane H^+^-ATPases was delayed and required a higher *C*_i_ under red light compared with in darkness (Ando *et al.*, 2022). These results also suggest that red light-induced phosphorylation of plasma membrane H^+^-ATPases is a component of the *C*_i_-independent response (Ando *et al.*, 2022).

The CA1 and CA4 deficiencies are known to impair the *C*_i_-dependent stomatal closure response. The diminished high *C*_i_ response in *ca1ca4* also explains the reduced *C*_i_ sensitivity of *g*_s,int_ since stomata remain more open, even under dark conditions (Figs 2B, 4A). The absence of a *C*_i_-dependent *g*_s,int_ response could also suggest that β-CA activity, downstream HCO_3_^−^ accumulation and sensing, or both is required for the induction of low *C*_i_-induced stomatal opening in the absence of light. Impairment of low *C*_i_-induced stomatal opening in this mutant is consistent with previous results, in which the response of *ca1ca4* to low CO_2_ was delayed and less pronounced (Hu *et al.*, 2010; Matrosova *et al.*, 2015). Matrosova *et al.* (2015) also suggest that the *C*_i_-independent red light response is stronger than the response to low CO_2_ in *ca1ca4*. Although *C*_i_ was not controlled in their experiments, this suggestion is consistent with our observations that a concomitant light signal is required to evoke an opening response to low *C*_i_ in these CO_2_ hyposensitive plants, as low *C*_i_ failed to induce stomatal opening in the dark (Fig. 4A). Crucially, the inclusion of *ca1ca4* in our experiments enabled further isolation of *C*_i_-independent stomatal responses from the *C*_i_-dependent pathway. In particular, suppression of high *C*_i_-induced stomatal closure in *ca1ca4* removed the interaction between *C*_i_ and Q_A_ redox state on the *g*_s_ response, giving rise to an additive model in which the slope of each regression line was similar across all *C*_i_ values (Supplementary Table S5; Figs 3B, 4B, 6). As such, the *C*_i_-independent and *C*_i_-dependent mechanisms of stomatal control, as estimated by Q_A_ redox state and *C*_i_, respectively, appear to independently coordinate *g*_s_ in *ca1ca4* (Figs 3B, 4B, 6).

### What signalling pathways coordinate the *C*_i_*-*dependent and -independent responses?

Overall, it seems that while the signalling pathways of the *C*_i_*-*dependent and -independent stomatal red light responses partially overlap, other components of these pathways are likely distinct. Components such as HT1, CBC1*/*2, and SLAC1 appear likely candidates for crosstalk between the two pathways as mutants display perturbed responses to both red light and CO_2_ (Laanemets *et al*., 2013; Matrosova *et al.*, 2015; Hiyama *et al.*, 2017). Furthermore, experiments by Ando and Kinoshita (2018) showed that while reductions in *C*_i_ promoted plasma membrane H^+^-ATPase activation in red light-illuminated samples, low *C*_i_ in darkness failed to induce phosphorylation required for activation, demonstrating the need for both stimuli. Interestingly, in isolated epidermal peels the combination of low *C*_i_ and red light illumination also failed to induce stomatal opening, suggesting a role for a much-debated mesophyll-derived signal (see review by Lawson *et al*., 2014).

In contrast to the shared signalling components above, a number of molecular players appear to have a distinct role in the *C*_i_-dependent pathway. For example, several CALCIUM DEPENDENT PROTEIN KINASES (CPKs) appear to exclusively respond to CO_2_; the *cpk3/5/6/11/23* quintuple mutant showed impaired stomatal responses to high and low CO_2_, but stomatal opening in response to red light was unaffected (Schulze *et al.*, 2021). Likewise, in the double carbonic anhydrase *ca1ca4* mutant the stomatal response to CO_2_, but not red light, was diminished (Matrosova *et al.*, 2015; Ando *et al.*, 2022). Interestingly, plasma membrane H^+^-ATPase phosphorylation and stomatal opening under red light were both increased in *ca1ca4*, compared with wild type (Ando *et al.*, 2022), which may imply that plants could compensate for the deficiency in the *C*_i_-dependent pathway by enhancing the *C*_i_-independent pathway to maintain effective carbon gain.

It is more difficult to pin-point the molecular determinants that are involved in sensing and communicating the red light stimulus in the *C*_i_-independent pathway. By isolating the *C*_i_-independent responses across a range of *C*_i_ values, we could establish strong linear correlations between Q_A_ redox state and *g*_s_ (Fig. 5; Supplementary Table S4), with similar regression slopes across ambient and sub-ambient *C*_i_ values (Fig. 5). The maintenance of this positive linear correlation across all measured *C*_i_ values in *ca1ca4* (Fig. 6; Supplementary Table S5) provides further support for the inclusion of PQ redox state as a predictor of red light-induced stomatal movements. These results are consistent with the hypothesis that PQ redox state, which reflects the balance between the excitation pressure at PSII and Calvin cycle activity, could provide an early signal to coordinate *C*_i_-independent responses (Busch, 2014). The involvement of PQ redox state is also consistent with the abolishment of red light-induced opening by DCMU, which blocks electron transfer upstream of PQ, maintaining an oxidized PQ pool (Sharkey and Raschke, 1981; Olsen *et al.*, 2002; Messinger *et al.*, 2006; Wang *et al.*, 2011; Ando and Kinoshita, 2018), and observed stomatal responses in *PsbS* overexpression and silenced *N. tabacum* lines (Głowacka *et al.*, 2018), which show a perturbed PQ redox state via altered excitation pressure at photosystem II. Though it seems PQ redox state could provide an early cue in the stomatal red light response, all empirical evidence accumulated thus far is largely correlative, making establishment of cause and effect difficult.

### What cell types coordinate *C*_i_-dependent and -independent responses?

The cellular origin of the stomatal red light response has been strongly contested, particularly when the response is partitioned into the *C*_i_-dependent and -independent components. Within the *C*_i_-dependent response, despite advances characterizing the pathway components that govern stomatal responses to CO_2_, evidence for whether the response is GC-autonomous or is also affected by signals from the underlying mesophyll remains unclear. For example, wild-type stomatal behaviour via GC-specific complementation of *ca1ca4* with either CA1 or CA4 (Hu *et al.*, 2010) suggests the response to *C*_i_ is largely GC-autonomous and perhaps only indirectly influenced by mesophyll photosynthesis via changes in *C*_i_. In the reaction–diffusion model developed by Tholen and Zhu (2011), the exclusion of CA activity substantially reduced predictions of mesophyll conductance, but net CO_2_ assimilation rate was largely unaffected. Thus, although CA1 and CA4 are highly expressed in both GCs and mesophylls cells (Leonhardt *et al.*, 2004; Yang *et al.*, 2008; Hu *et al.*, 2010) any CA-mediated control of stomatal behaviour likely predominates within the GCs. However, studies using isolated GCs to delineate the cellular origin of the *C*_i_-dependent response also provide conflicting evidence. For example, stomatal CO_2_ responses were reduced in isolated epidermal samples, but could be rescued via reattachment to the mesophyll (Mott *et al.*, 2008; Fujita *et al.*, 2013). Similarly, the use of diffusional barriers between GCs and mesophyll cells impaired the stomatal response to CO_2_ (Fujita *et al.*, 2013). Although these studies propose a more direct role for mesophyll-derived signals in coordinating stomatal responses to *C*_i_, it also seems important to consider the potential mechanical role mesophyll cells could play in modulating GC behaviour. For example, it is well established that mesophyll cells translocate solutes across the GC plasma membrane, such as sucrose and the organic anion malate (Lu *et al.*, 1995, 1997; Ritte *et al.*, 1999; Outlaw and De Vlighere-He, 2001; Roelfsema and Hedrich, 2005; Medeiros *et al.*, 2017; Flütsch and Santelia, 2021), which could directly modulate GC turgor by altering their osmotic potential, but also via the involvement and recruitment of downstream sucrose or malate sensing and signal transduction pathways (Lawson *et al.*, 2014; Santelia and Lawson, 2016). In addition, the need for underlying mesophyll tissue as a prerequisite for GC responses to CO_2_ is not consistently observed (Edwards and Bowling, 1985; Brearley *et al.*, 1997; Webb and Hetherington, 1997; Hu *et al.*, 2010).

For *C*_i_-independent stomatal behaviour, even less is understood regarding its cellular origin. For example, it remains unclear whether the putative chloroplast-derived PQ signal would originate from the mesophyll, requiring further transduction to the GCs (Roelfsema *et al.*, 2002; Lawson *et al.*, 2014), or within the GCs themselves (Zeiger *et al*., 1983; Olsen *et al.*, 2002). The fluorescence measurements implemented here and by Głowacka *et al.* (2018) to estimate Q_A_ redox state (Figs 1F, 3F) predominantly represent mesophyll cell chloroplasts. However, the fact that guard cells almost ubiquitously contain chloroplasts (Shimazaki and Zeiger, 1985; Cardon and Berry, 1992; Tsionsky *et al.*, 1997; Lawson *et al.*, 2002, 2003; Lawson, 2009), which behave similarly in response to light and CO_2_ stimuli compared with mesophyll chloroplasts (Lawson *et al.*, 2002, 2003), implies that the observed Q_A_ redox state patterns could be highly correlated between both cell types. If so, the parsimony principle would favour a cell-autonomous signalling pathway derived locally from the Q_A_ redox state of the guard cell chloroplasts, which would remove the need for intercellular signal transduction. To provide answers to these questions the measurement protocol used here should be used to study lines with cell type-specific manipulations of stomatal response pathways.

### Conclusions

Measurement of *g*_s_ as a function of a matrix of *C*_i_ values and red light intensities allowed easy isolation of *C*_i_-dependent and -independent mechanisms in coordinating stomatal red light responses, and for the first time, provided a quantitative assessment of the relative magnitude of each pathway. While both pathways were found to contribute equally to red light-induced stomatal opening, the suppression of *C*_i_-independent opening at high *C*_i_ demonstrates interaction between the two pathways. The maintenance of stomatal opening across all *C*_i_ values in the CO_2_ hyposensitive mutant *ca1ca4* removed the interaction between *C*_i_ and Q_A_ redox state, giving rise to an additive model, suggesting the *C*_i_-dependent and -independent components are distinct and jointly coordinate the overall stomatal response to red light. Finally, our findings expand the range of conditions under which the linear relationship observed between *g*_s_ and Q_A_ redox state is maintained and show that this correlation is specific to the *C*_i_-independent component of the stomatal red light response. While these results are consistent with the putative PQ redox signal coordinating the *C*_i_-independent component, the correlative nature highlights the need for future research to further characterize the signalling components and specific cell types involved in coordinating *C*_i_-independent stomatal red light responses.

## Supplementary data

The following supplementary data are available at *JXB* online.

Fig. S1. Determining the response of *C*_i_ at a range of physiologically relevant red light intensities.

Table S1. Genotyping primers used in this study.

Table S2. Dunn’s pairwise comparison of the initial dark conductance (*g*_s,int_) and the total stomatal response (Δ*g*_s_) in Arabidopsis Col-0.

Table S3. Dunn’s pairwise comparison of the initial dark conductance (*g*_s,int_) and the total stomatal response (Δ*g*_s_) in the CO_2_ hyposensitive mutant *ca1ca4.*

Table S4. Multiple linear regression analysis of the relationship between *g*_s_ and Q_A_ redox state in Col-0.

Table S5. Multiple linear regression analysis of the relationship between *g*_s_ and Q_A_ redox state in *ca1ca4*.

erae093_suppl_Supplementary_Tables_S1-S5_Figure_S1

## Data Availability

All data supporting the findings of this study are available within the paper and within its supplementary materials.
