# Supplementary material for: Illuminating stomatal responses to red light: establishing the role of Ci-dependent versus -independent mechanisms in control of stomatal behaviour
Source: J Exp Bot. 2024 Mar 5;75(21):6810–22. doi: 10.1093/jxb/erae093 (PMC11565200; doi:10.1093/jxb/erae093)
Supplement: erae093_suppl_Supplementary_Tables_S1-S5_Figure_S1 [file erae093_suppl_supplementary_tables_s1-s5_figure_s1.pdf]

Table S1. Genotyping primers used in this study.

**Primer**	**Sequence (5’-3’)**
*ca1*_LP	CTTCGTGGTCCGTAACATAGC
*ca1*_RP	ATCGGGTAAGGCTTCAAAGAG
*ca4*_LP	GGAAAAAGACGAAATGGCAAC
*ca4*_RP	GAGCAAGCAAACACCAGAAAC
LB1.3	ATTTTGCCGATTTCGGAAC

Table S2. Dunn's pairwise comparison of the initial dark conductance ($g_{s\ int}$) and the total stomatal response (Δg_s) in *A. thaliana* Col-0.

Response	Comparison (C_i concentration)	*P. adj*	*P. adj.sig*
$g_{s\ int}$	75 – 150	1	ns
	75 – 300	0.409	ns
	75 – 375	0.00234	**
	75 – 460	0.00611	**
	75 – 600	0.000402	***
	75 – 750	0.0106	*
	150 – 300	0.897	ns
	150 – 375	0.0102	*
	150 – 460	0.0226	*
	150 – 600	0.00202	**
	150 – 750	0.0330	*
	300 – 375	1	ns
	300 – 460	1	ns
	300 – 600	1	ns
	300 – 750	1	ns
	375 – 460	1	ns
	375 – 600	1	ns
	375 – 750	1	ns
	460 – 600	1	ns
	460 – 750	1	ns
	600 – 750	1	ns
Δg_s	75 – 150	1	ns
	75 – 300	1	ns
	75 – 375	1	ns
	75 – 460	1	ns
	75 – 600	0.10316	ns
	75 – 750	0.02496	**
	150 – 300	1	ns
	150 – 375	1	ns
	150 – 460	0.54006	ns
	150 – 600	0.04423	*
	150 – 750	0.01056	**
	300 – 375	1	ns
	300 – 460	0.03868	*
	300 – 600	0.00139	**
	300 – 750	0.00031	***
	375 – 460	1	ns
	375 – 600	0.08504	ns
	375 – 750	0.02007	*
	460 – 600	1	ns
	460 – 750	1	ns
	600 – 750	0.02496	*

Table S3 Dunn's pairwise comparison of the initial dark conductance ($g_{s\ int}$) and the total stomatal response (Δg_s) in the CO_2 hyposensitive mutant *ca1ca4*

Response	ANOVA Results	Comparison (C_i concentration)	*P. adj*	*P. adj.sig*
$g_{s\ int}$	*df* = 6<br>*F-value* = 1.072<br>*P* = 0.394	NA	NA	NA
Δg_s	*df* = 6<br>*F-value* = 3.98<br>*P* = 0.00281	75 – 150	0.93684	ns
		75 – 300	0.32837	ns
		75 – 375	0.01574	*
		75 – 460	0.01898	*
		75 – 600	0.02455	*
		75 – 750	0.01570	*
		150 – 300	0.91925	ns
		150 – 375	0.94653	ns
		150 – 460	0.23638	ns
		150 – 600	0.28040	ns
		150 – 750	0.20369	ns
		300 – 375	0.82327	ns
		300 – 460	0.88569	ns
		300 – 600	0.91983	ns
		300 – 750	0.85194	ns
		375 – 460	1	ns
		375 – 600	1	ns
		375 – 750	1	ns
		460 – 600	1	ns
		460 – 750	1	ns
		600 – 750	1	ns

Table S4. Multiple linear regression analysis of the relationship between g_s and Q_A redox in Col-0

**FACTOR**	**Slope**	**std.error**	**T-value**	***P.adj***
CONTROLCI_150	0.163839	0.007945	20.621	< 2e-16 ***
QA	0.110206	0.014843	7.425	8.09e-13 ***
CONTROLCI_300	-0.064425	0.010813	-5.958	6.03e-09 ***
CONTROLCI_375	-0.082985	0.010531	-7.880	3.82e-14 ***
CONTROLCI_460	-0.087674	0.010706	-8.190	4.49e-15 ***
CONTROLCI_600	-0.104039	0.010681	-9.741	< 2e-16 ***
CONTROLCI_75	0.031363	0.010920	2.872	0.00432 **
CONTROLCI_750	-0.094641	0.011445	-8.269	2.56e-15 ***
QA:CONTROLCI_300	0.011642	0.020913	0.557	0.57807
QA:CONTROLCI_375	-0.013298	0.020549	-0.647	0.51795
QA:CONTROLCI_460	-0.046701	0.021717	-2.150	0.03218 *
QA:CONTROLCI_600	-0.062092	0.021491	-2.889	0.00409 **
QA:CONTROLCI_75	-0.016024	0.019248	-0.833	0.40566
QA:CONTROLCI_750	-0.091762	0.023212	-3.953	9.27e-05 ***

Table S5. Multiple linear regression analysis of the relationship between g_s and Q_A redox in *ca1ca4*

**FACTOR**	**Slope**	**std.error**	**T-value**	***P.adj***
Intercept	1.597e-01	6.119e-03	26.094	< 2e-16 ***
QA	1.956e-01	1.421e-02	13.765	< 2e-16 ***
CONTROL	-5.716e-05	1.089e-05	-5.247	2.64e-07 ***

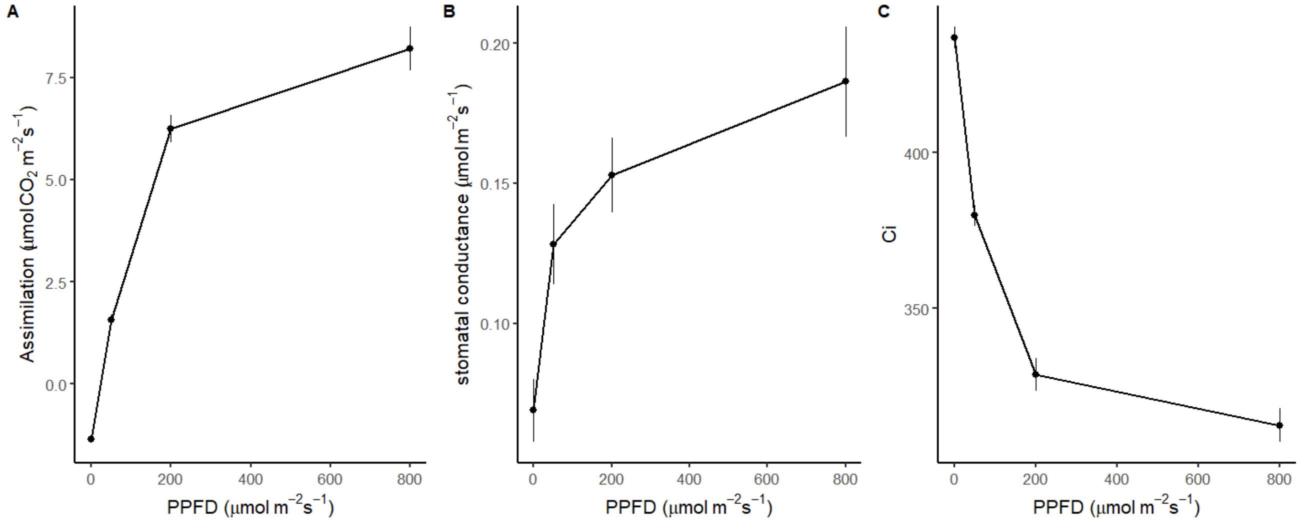

Figure S1. Determining the response of *Ci* for *Arabidopsis* Col-0 at a range of physiologically relevant red-light intensities. The response of **(a)** CO_2 assimilation, **(b)** stomatal conductance and **(c)** Intercellular CO_2 concentration (*Ci*) at dark (D), low-light (LL), growth light (GL) and high-light (HL) intensities under ambient CO_2 (410 ppm). n=5.
